# Are Competition and Extrinsic Motivation Reliable Predictors of Academic Cheating?

**DOI:** 10.3389/fpsyg.2013.00087

**Published:** 2013-02-27

**Authors:** Gábor Orosz, Dávid Farkas, Christine Roland-Lévy

**Affiliations:** ^1^Institute of Psychology, University of SzegedSzeged, Hungary; ^2^Department of Psychology, University of Rheims Champagne-ArdenneRheims, France; ^3^Institute of Cognitive Neuroscience and Psychology, MTA Research Centre for Natural Sciences, Hungarian Academy of SciencesBudapest, Hungary; ^4^Department of Cognitive Science, Faculty of Natural Sciences, Budapest University of Technology and EconomicsBudapest, Hungary

**Keywords:** academic cheating, competition, intrinsic motivation, extrinsic motivation, amotivation, competitive climate, hypercompetition, self-developmental competition

## Abstract

Previous studies suggest that extrinsic motivation and competition are reliable predictors of academic cheating. The aim of the present questionnaire study was to separate the effects of motivation- and competition-related variables on academic cheating by Hungarian high school students (*N* = 620, *M* = 264, *F* = 356). Structural equation modeling showed that intrinsic motivation has a negative effect, and amotivation has a positive indirect effect on self-reported academic cheating. In contrast, extrinsic motivation had no significant effect. Indirect positive influence on cheating, based on some characteristics of hypercompetition, was also found, whereas attitudes toward self-developmental competition had a mediated negative influence. Neither constructive nor destructive competitive classroom climate had a significant impact on academic dishonesty. Acceptance of cheating and guilt has significant and direct effect on self-reported cheating. In comparison with them, the effects of motivational and competition-related variables are relatively small, even negligible. These results suggest that extrinsic motivation and competition are not amongst the most reliable predictors of academic cheating behavior.

## Introduction

### Competition in the classroom

In the introduction of Anderman and Murdock’s (2007) seminal book on academic cheating, the authors summarized the role of classroom competition in the following way: “*Competition is perhaps the single most toxic ingredient in a classroom, and it is also a reliable predictor of cheating*” (Anderman and Murdock, 2007, p. XIII). According to several former studies and reviews (Lewis, 1944; Lewis and Franklin, 1944; Deutsch, 1949; Johnson and Johnson, 1974, 1979, 1982; Johnson et al., 1981; Qin et al., 1995) competition has an overall negative outcome on performance, problem solving, and personal relationships as compared to cooperation. Furthermore, several previous studies suggest that competition undermines intrinsic motivation (Deci et al., 1981; Vallerand et al., 1986). Between 1940 and 1990, few studies reported positive aspects of competition (Julian et al., 1966; Rabbie and Wilkens, 1971; Vallerand and Reid, 1984; Reeve et al., 1986). However, both before and after this period several articles have suggested that competition can also have positive effects on performance, interpersonal relationships, resource control, intrinsic and extrinsic motivations, etc. (Hurlock, 1927; Sims, 1928; Reeve et al., 1985; Bornstein et al., 1990; Wentzel, 1991; Epstein and Harackiewicz, 1992; Erev et al., 1993; Young et al., 1993; Reeve and Deci, 1996; Ryckman et al., 1996; Tassi and Schneider, 1997; Harackiewicz et al., 1998; Fülöp, 1999, 2001, 2004; Hawley, 2003, 2006; Tjosvold et al., 2003, 2006; Tauer and Harackiewicz, 2004). For example, Erev et al. (1993) found that intergroup competition lead to higher performance in an orange picking task than in individual or cooperative settings. Tjosvold et al. (2003, 2006) found that the constructive competition (CC) exist in organizational context if employes see fairness in the process of competition. In an educational context, Fülöp (1999, 2004) demonstrated that Japanese students see competition in a positive manner. According to them the main functions of competition are the development and motivation. Japanese students are mainly oriented toward self-development during competitions. In sum, a significant amount of research have demonstrated that competition can have positive consequences; therefore, it can be useful to distinguish at least the two main forms of it, namely its constructive and destructive aspects (Erev et al., 1993; Tjosvold et al., 2003, 2006; Fülöp, 2008).

*Constructive competition* occurs when competition is a positive, enjoyable experience resulting in increased efforts to achieve, more positive interpersonal relationships, and greater psychological health and well being (Tjosvold et al., 2003, p. 65).

Whereas, according to Fülöp (2008), *destructive competition* (DC) is harmful at least for one of the competitors. Moreover, in this type of competition, rivals frequently experience anger and envy; communication between adversaries becomes distorted by dishonesty and lack of trust. Regarding the outcomes of the competition, the winner’s self-enhancement motives become dominant and he/she gloats over the loser, whereas the loser often accuses the winner of cheating and of being dishonest, then he/she quits the situation and tries to be away from further competitive situations; in this latest case self-improving and learning motives are lacking.

In the literature on academic cheating, competition was shown to be in positive relationship with cheating (Smith et al., 1972; Whitley, 1998). For example, Taylor et al.’s (2002) study showed that the main reason for cheating in elite high schools was the great competitive pressure to get good grades. In sum, the majority of the results suggested that competition leads to several negative outcomes within and beyond the classroom and competition is regarded as a factor that facilitates academic dishonesty (Smith et al., 1972; Whitley, 1998; Taylor et al., 2002; Anderman and Murdock, 2007). Therefore, it might be interesting to test that not all forms of competition are in a positive relationship with the occurrence of cheating. From practical perspectives, this might be a relevant question as it would be beneficial to create competitive educational environments that do not induce cheating and other negative outcomes but potential higher performance, e.g., good grades (Harackiewicz et al., 1998). To our best knowledge, no previous study investigated the effect of individual- or situational-level constructive vs. destruction competition on academic dishonesty.

Several questions arise concerning the relationship between academic cheating and competition. In the present study we intend to assess the impact of individual level and situation-level competition-related variables on academic cheating. Furthermore, we aim to examine the magnitude of these effects in comparison with other individual and situational variables which can influence the prevalence of cheating. In the following section, previous findings about the utilized (a) individual, (b) situational and contextual predictors of the present study will be reviewed. Then, specific societal issues, which may influence academic cheating in Hungary, will be described.

### Individual predictors of cheating

#### Individual predictors

In the present study, we examined the roles of attitudes toward competition, grade point average (GPA), attitudes toward cheating, guilt, as well as academic motivation as individual factors affecting cheating behavior. According to Whitley’s (1998) meta-analysis, and other studies (Smith et al., 1972; Taylor et al., 2002; Anderman and Murdock, 2007), classroom competition is in a positive relationship with academic cheating. However, these studies focused mainly on the impact of classroom competition on cheating, and not on individual differences regarding attitudes toward competition. Among the individual factors related to competition, it is possible to define *self-developmental* (Ryckman et al., 1996) competitors, who focus on their own personal development, do not perceive their adversaries as enemies, and enjoy the process of competition, because they can learn from it. Beyond these two dimensions we can suppose a third factor also which refers to general positive attitudes toward competition (PAC). It can be defined as the preference of competitive situations and competitive challenges. A previous study (Orosz, 2010) showed that self-developmental (SD) competition and collaborative cheating which can be defined according to McCabe et al., 2001, p. 221) as “*unpermitted collaboration among students on written assignments*” are in a negative relationship with each other. Ryckman et al. (1990, 1996) distinguished hypercompetitive traits from self-developmental characteristics. *Hypercompetitive* individuals strive to win at any cost; they see their rival as enemies and can be aggressive toward them. Based on Ryckman et al.’s (1990, 1996) work, a questionnaire was created for the current study, in order to distinguish three main individual factors involved in competition: (1) hypercompetition (HC), which is expected to be in a positive relationship with cheating; (2) SD competition, which is assumed to be in a negative relationship with cheating, and (3) general PAC, which is hypothesized to be unrelated to cheating.

Earlier results (Leming, 1978; Kerkvliet, 1994; Newstead et al., 1996; Whitley, 1998; Kerkvliet and Sigmund, 1999; Straw, 2002) showed a negative relationship between academic cheating and GPA. These studies found that students, who had higher GPA, cheated less during their assignments than their peers with lower GPA. In our study, we expect GPA to be in a negative relationship with cheating.

Whitley’s (1998) meta-analysis, covering 74 studies, as well as other more recent studies (Jordan, 2001; Jensen et al., 2002; Bolin, 2004) showed that *positive attitudes toward cheating* have a very important impact on actually committing cheating in school. Therefore, we hypothesize that attitudes regarding how acceptable a student finds cheating will prove to be a strong predictor of self-reported cheating behavior.

Malinowski and Smith (1985) found negative relationship between the feeling of *guilt* and cheating; Diekhoff et al. (1999) showed that among American and Japanese students who do not cheat, guilt is the most effective deterrent. Consequently, we expect that guilt will show an inverse relationship with academic dishonesty.

On the theoretical basis of Deci and Ryan’s (1985) Self Determination Theory, and using Vallerand et al.’s (1992) Academic Motivation Scale (AMS) we aimed to take into consideration three forms of academic motivational regulations: intrinsic motivation, extrinsic motivation, and amotivation (AM). *Intrinsic* motivation refers to doing an activity for its own sake and for the pleasure and satisfaction deriving from it. Vallerand et al. (1992) defined three subcomponents of intrinsic motivation. (a) The *intrinsic motivation to know* can be defined as performing an activity for the pleasure and the satisfaction during learning or during the exploration of a new thing. (b) The *intrinsic motivation toward accomplishment* appears when a student focuses on the process of achieving rather than on the outcome. (c) The *intrinsic motivation to experience stimulation* refers to engaging in an activity for the stimulating experiences, such as esthetics, sensory pleasure or fun. *Extrinsic* motivation appears when an individual is engaged in an activity, not for its own sake but as a means to an end. It can also be separated into three subtypes. (a) Initially, an *external regulation* determines the behavior, in terms of rewards or constraints. (b) Later, with *introjected regulation*, the individual starts to internalize the reasons of his/her behavior, but it still mainly depends on its external effects. (c) Finally, *identification* refers to a motivation when the behavior becomes important and he/she feels that the activity was self-selected. When an individual does not perceive causality between his/her actions and their results, this can be labeled as AM. Individuals with AM have neither extrinsic nor intrinsic motivations, and they typically feel incompetent regarding the given activity/field, and sooner or later they may not participate in academic activities. Students who are characterized by AM feel that their school-related activity is out of their own control (Vallerand et al., 1992).

Studies examining the correspondence between school-related motivations and cheating found that extrinsic motivation, performance goal, or grade orientation is in a positive relationship with cheating behavior. Performance-oriented students seek recognition for their achievements; they wish to demonstrate and validate their competences by seeking positive judgments and avoiding negative opinions about their competences; therefore, they strive to achieve well on external indicators of success (i.e., grades). Whereas students with high mastery goal orientation are involved in school-related tasks for the sake of self-development during learning; they wish to become proficient in a given topic, and they focus mainly on the development of competences. In contrast, intrinsic motivation and mastery goal orientation were in a negative relationship with cheating (Weiss et al., 1993; Anderman et al., 1998; Pulvers and Diekhoff, 1999; Wryobeck and Whitley, 1999; Jordan, 2001; Murdock and Anderman, 2006). According to Anderman et al.’s (1998) and Jordan’s (2001) results, students who behaved honestly during exams and other assignments were characterized by high intrinsic and low extrinsic motivation. Furthermore, Pulvers and Diekhoff (1999) found that students who evaluated the class topics as interesting cheated less. According to Murdock and Anderman (2006) review, middle school students, who did not report cheating, had a higher level of mastery of goals than those who self-confessed cheating. Other studies showed that strong grade orientation – which belongs to the category of extrinsic motivation – is in a positive relationship with cheating (Weiss et al., 1993). On the basis of the review carried out by Murdock and Anderman (2006), regarding both individual goal structure and classroom goal structure, extrinsic forms of motivation are associated with cheating, whereas intrinsic motivations is associated with honest behavior in students. In sum, according to previous results, extrinsic motivation appears to have a positive effect on academic cheating, whereas intrinsic motivation has a negative effect on it. Consequently, we expect that intrinsic motivation will show a positive relationship with academic dishonesty; extrinsic motivation will be in a weak positive relationship with cheating; whereas AM will be in a stronger positive relationship with cheating.

### Situational and contextual predictors of cheating

#### Situational and contextual variables

Both Whitley’s (1998) meta-analysis and McCabe and Trevino’s (1997) large scale study suggested that contextual variables have a greater impact on cheating behavior than individual factors. Among the numerous contextual variables, the role of risk of detection and expected punishments were examined in our study. Various studies found that the perceived *risk of detection* – the probability of being caught – was inversely related with cheating behavior (Heisler, 1974; Leming, 1978; Corcoran and Rotter, 1987; Covey et al., 1989; Whitley, 1998). Furthermore, in Becker’s (1968) economic model, and in tax fraud literature (Kirchler, 2007), risk of detection was regarded as a factor, which can reduce the prevalence of dishonesty. According to Title and Rowe’s (1973) results *punishments* can be useful deterrents of academic cheating. However, previous studies that examined American (Bunn et al., 1992; Cohran et al., 1999), Japanese (Diekhoff et al., 1999), UK (Salter et al., 2001), and Lebanese students (McCabe et al., 2008) suggested that punishment might not be the most optimal tool for reducing the occurrence of academic dishonesty. Bunn et al. (1992), for example, found that the expected gravity of punishment was unrelated to students’ cheating. In line with these results, Cohran et al. (1999) also failed to find any deterring effect the threat of formal sanctions on academic dishonesty. On the other hand, McCabe and Trevino (1993) reported that social variables, such as seeing other students cheating and acquaintance of a classmate who regularly cheated, were related to academic dishonesty. Consequently, we expect that the risk of detection and the gravity of punishment will be in a negative relationship with cheating.

### Societal and educational system level predictors of cheating

Cheating behavior can be influenced by distal influences originating from differences in the educational systems and societies in which students are embedded. In Eastern-European countries, the prevalence of academic cheating is 87.9%; this number is surprisingly high in comparison with approximately 5% measured in Scandinavian countries (Teixeira and Rocha, 2010). Furthermore, on the basis of Grimes (2004) results, in post-socialist countries, the number of students who self-report cheating is significantly higher than in the USA. Poltorak (1995) found that Russian students, even most of those who regard academic dishonesties as cheating, find assignment-related (mainly collaborative) dishonesties acceptable. The author explains this by the pervasive presence of cheating at the societal-level. She argues that, due to the egalitarian ideology of the socialist era, Russian people got used to the lack of competition, leading to collaboration with other people. Furthermore, the majority of the society did not identify with the communist ideology, which created reluctance to cooperate with the authorities. This opposition with authorities can lead to the perception of legitimization of cheating. These conditions can be amongst the reasons why cheating, especially in collaborative forms, is committed so frequently by students from Moscow. Consequently, based on Poltorak’s (1995) sociological perspective, societal-level analysis is relevant for explaining academic dishonesty. In sum, on the basis of previous studies (Poltorak, 1995; Lupton et al., 2000; Magnus et al., 2002; Grimes, 2004; Hrabak et al., 2004; Teixeira and Rocha, 2006, 2010; Orosz, 2009), academic cheating in the Eastern-European region, appears to be a more serious issue than in Western-Europe or in North-America.

Beyond societal-level factors, according to Poltorak (1995), high prevalence of academic cheating in Moscow is also rooted in the malfunctioning education system. She found that Russian students rationalize their cheating behavior by accusing the educational system. In the communist period, educational institutions were the most important distributors of the communist ideology. Therefore, the curriculum was permeated by ideology-based topics. However, the overall ideology was not accepted by the majority of Russians. Therefore, students viewed cheating as an act against the authorities (such as teachers) who propagated the communist ideology and, therefore, cheating became a justified and acceptable act for them. Another factor underlying the high occurrence of cheating amongst students in Moscow was the low level of competition, which promoted collaborative cheating behavior. As we already described, in other cultures, researches on academic dishonesty assess the role of competition differently (e.g., Anderman et al., 1998; Levitt and Dubner, 2005; Anderman and Murdock, 2007; Nichols and Berliner, 2007). Here, we note that competition may play a smaller and, possibly, a different role in the educational systems of post-socialist countries.

### Goals and hypotheses

The present study has three main goals. First (H1), we assume that competition has a multifaceted impact on cheating behavior. We expect one of them to be that hypercompetitive students will cheat more. However, students who aim to develop themselves through competition (Ryckman et al., 1996) and those who have PAC would cheat less. As for situational variables, here we consider the classroom atmosphere in relation to educational competition. We hypothesize that in classes in which competition activates extrinsic motivations as achieving recognition by the teachers, prevalence of cheating will be higher^1^. In contrast, classroom environments in which students enjoy competition and competitive skill development is promoted do not increase the occurrence of cheating. Thus, we aim to rearticulate the explanation of the effect of competition on cheating behavior, both at the individual and at the contextual level.

Our second goal (H2) is to distinguish the effects of motivation vs. competition-related factors of cheating. We based our second hypothesis on the reexamination of several previous studies (Anderman et al., 1998; Anderman and Midgley, 2004; Murdock and Anderman, 2006; Anderman and Murdock, 2007) which found that both mastery and intrinsic motivations is negatively related to cheating, while performance goals and extrinsic motivations are in a positive relationship with it. However, these studies did not treat competitive pressures and performance-related goal orientation or extrinsic motivation as possibly separate factors, but as interconnected and overlapping constructs. Hence, it remains unclear whether motivational and competition-related factors can separately influence cheating. This distinction becomes even more relevant if competition is considered as a multidimensional concept, which can have both negative and positive effects on cheating behavior. Therefore, we hypothesize that the effects of the motivational variables (i.e., intrinsic motivation, extrinsic motivation, and AM) are distinct from the competition-related individual and contextual variables.

Finally, complementing the work of Anderman and Murdock (2007), our third goal is to compare the relative importance of motivational and competition-related variables with other variables involved in cheating behavior, such as attitudes toward cheating, guilt, risk of detection, and possible punishments. To this end, demographic, individual (GPA, attitudes toward cheating, guilt), situational, and interpersonal (risk of detection, expected punishments) variables were also assessed. If motivational and competition-related issues end up being relevant and reliable predictors of cheating, then they should show strong direct effects on self-reported cheating behavior. Otherwise, it is possible that the importance of motivational and competition-related factors is overrated in the literature of academic cheating.

We hypothesize (H3) that the magnitude of the effects of motivational and competition-related variables on academic dishonesty is significantly lower than those of individual (attitudes toward cheating, guilt) and of situational variables (risk of detection, perceived seriousness of punishments in case of being caught). We expect that academic motivations and competition are not among the most reliable predictors of cheating. In order to test this hypothesis firstly correlation coefficients of the relevant variables were compared, subsequently a path model was created which summarize the relationship pattern of the examined variables.

Finally, our study aimed at setting up an exploratory model for academic cheating, which takes into consideration both the individual (attitudes toward cheating, guilt, competition-related personality traits, learning motivation) and the situational interpersonal factors (risk of detection, expected punishments, and competitive climate).

## Materials and Methods

### Participants

Six hundred twenty high school students (*M* = 264, *F* = 356), from 19 classes in seven Hungarian high schools, participated in the study. The respondents’ age was between 13 and 20 years; the average age was 16.66 years old (SD = 1.51). Regarding the education level of parents, 2.4% of mothers have a primary level of education, 65.8% the secondary-level, while 31.8% of the mothers have a college or a university degree. Concerning the fathers, 2.8% have a primary level of education, 65.8% a secondary-level, and 31.4% have a higher-education degree. Participants were informed about the content of the questionnaire, e.g., competition, motivation, and academic cheating. Respondents volunteered for the study and students did not receive compensation for the participation. The schools and parents (passive consent) were informed about the topic of the research. Furthermore, students were assured of their anonymity and that teachers will not be informed about their responses. Questionnaires were filled in during class, where teachers were not present in the classroom; only the investigators were present during data-gathering. Students were asked to respond as honestly as possible. After filling the questionnaire, students were encouraged to give remarks and raise questions. Firstly, we intended to measure the relationship between cheating and individual differences in terms of competition and motivation. However, after the first data-gathering period, we included questions regarding situational factors of competitive climate. Consequently, of the 620 participants, 381 filled in the additional questionnaire, which contained items regarding competitive climate; while the remaining participants (*N* = 236) only filled in the initial questionnaire. There was no student who refused to participate.

### Variables and measures

The questionnaire was created specifically for testing our three hypotheses. On the first page, demographic data, such as gender, age, number of siblings, and qualifications of parents, school, specialization, GPA from the last semester, sport and other extracurricular activities, were asked.

The next section measured individual differences in competition. On the basis of Ryckman et al.’s (1990, 1996) hypercompetitive and SD competition scales, three new scales were created. The first scale referred to HC, similar to Ryckman et al.’s (1990) dimension. However, in order to meet the requirement of construct validity (see below), in our measures aggression and conflict-related aspects of competition were relatively more emphasized than in the original Hypercompetitive Attitude Scale. This scale contained four items such as “*I can be aggressive with my rivals*” and “*I’m often in conflict with my opponents*.” The second scale was a modified and shortened version Ryckman et al.’s (1996) personal developmental competitive scale. This scale also included four items, such as “*Competition helps me to improve my skills*” and “*Competition brings the best out of me*”; it focused on the self-improving nature of competition. Finally, we created a scale measuring general PAC. The four items of this scale were like “*I like the challenge of competition*” and “*Competition inspires me*.” For each item, the respondents marked on a four-point Likert type scale (1 = it does not apply to me at all, 4 = it applies to me perfectly) how much it related to them. We term this the “individual differences in competition” scale (IDCS).

In the second section, perceived competitive school climate, which contained two dimensions, was aimed to be measured. The first referred to perceived CC atmosphere. Three items of this scale were like “*Competitive situations at school develop students’ skills*” or “*Students like competition at school*.” The second dimension concerned perceived DC climate, which also included three items such as (in my school) “*There is a strong competition for the recognition of teachers*” or (in my school) “*Competitive situations exhaust students*.” These items were rated on a six-point Likert scale regarding their school environment (1 = doesn’t correspond at all; 2 = doesn’t correspond; 3 = rather doesn’t correspond; 4 = rather corresponds; 5 = corresponds; and 6 = corresponds a lot). We term this the “competitive climate” scale (CCS).

In the next section, two vignettes about cheating were presented to the students. One described a situation in which a student uses a cheating sheet during some test; the other depicted a situation in which a student copies the answers from his/her classmates during exam. Participants were instructed to evaluate these vignettes on the following dimensions: (a) acceptance of this behavior (attitude toward cheating), (b) perceived risk of detection in the given situation, (c) feeling of guilt after such form of cheating (d) expected punishment in the case of detection. The answers were given again with the help of a four-point Likert scale (1 = totally unacceptable/not at all risky/not at all/nothing/warning, 4 = totally acceptable/very risky/very/severe/expelling, for the four thematic evaluations, respectively). The final question, for the vignettes, referred to whether the student did something similar at least once during the last semester (self-confessed academic cheating); students could answer by *yes* (1) or *no* (0).

The next section of the questionnaire contained Vallerand et al.’s (1992) AMS for high school samples. This scale was translated following the protocol of Beaton et al. (2000). This instrument originally includes seven factors. Three of the factors refer to intrinsic (IM: “*to know*” – TK, “*toward accomplishment*” – TA, “*experience stimulation*” – ES), another three to extrinsic motivation (EM: “*external regulation*” – ER, “*introjected regulation*” – IJ, “*identified regulation*” – ID), and one measures AM. Students were asked questions, such as “*Why are you going to school?*” and the response choices for these items were rated on a seven-point Likert scale from (1 = doesn’t correspond at all; 2–3 = corresponds a little; 4 = corresponds moderately; 5–6 = corresponds a lot; and 7: corresponds exactly). The items can be found in Vallerand et al. (1989, 1992). This scale was validated and tested in several countries and languages, with a variety of populations (Vallerand et al., 1989, 1993; Cokley et al., 2001; Fairchild et al., 2005; Grouzet et al., 2006; Barkoukis et al., 2008; Smith et al., 2010). Except for one study (Cokley et al., 2001), the seven-factor structure of Vallerand et al.’s (1989, 1992, 1993) original concept was supported. In Grouzet et al.’s study, only five factors (IM, ID, IJ, ER, AM), out of the seven were measured, and a five-factor structure emerged.

The study conducted in accordance with the Declaration of Helsinki. All procedures were carried out with the adequate understanding and consent of the participants and with the approval of University of Szeged.

### Data analysis

First, exploratory factor analyses (EFA) were carried out on IDCS, CCS, and AMS using SPSS for Windows 15.0.0. These were followed by confirmatory factor analyses (CFA) using AMOS 17.0. G*Power 3 was used for reporting statistical power and reliability was measured by Cronbach’s alpha.

Exploratory factor analyses were conducted with Maximum Likelihood (ML) extraction and promax rotation (Kappa = 4), because on one hand, this method provides a more realistic representation of how factors are interrelated, and on the other hand, ML oblique solutions are more likely to generalize to CFA than orthogonal solutions (Brown, 2006). In order to assess an appropriate number of factors, we took into account both the Guttman–Kaiser criterion (Guttman, 1954; Kaiser, 1960) and the scree test (Cattell, 1966). The Kaiser–Meyer–Olkin measure of sampling adequacy and Bartlett’s test of sphericity were used to confirm that the items were suitable for factor analysis. Taking into account all of the measured items missing data was 0.4% (*M* = 2.95 missing values/item, ranged between 0 and 14), which were substituted by means. Following Tabachnik and Fidell’s (2001) guidelines, the minimum loading of an item was set at 0.32 and “cross-loading” was interpreted as if an item load was at 0.32 or higher on two or more factors.

Confirmatory factor analyses and path analyses were conducted on covariance matrices, and the solutions were generated by ML estimation. Following Brown’s (2006) guidelines as well as Schreiber et al.’s (2006), several different indexes of goodness of fit were taken into consideration, including chi-square degree of freedom ratio (χ^2^/*df*), root mean square error of approximation (RMSEA), and its 90% confidence interval (90% CI), as well as tests of close fit (CFit), comparative fit index (CFI), and the Tucker–Lewis index (TLI). Guided by suggestions provided in Hu and Bentler (1999), acceptable model fit was defined by the following criteria: RMSEA (≤0.06, 90% CI ≤ 0.06, CFit *ns*), CFI (≥0.95), and TLI (≥0.95).

In the correlational and path analyses, self-reported cheating was measured in a dichotomous way (cheated or not) in two situations; therefore, this dependent variable was based on a three-point scale: 1. no cheating at all, 2. cheated in only one way – copying or using cheating sheets, 3. cheated in both ways.

## Results

Firstly, EFA then CFA and reliability results will be described in the following order: IDCS, CCS, and AMS (Vallerand et al., 1992). In the case of cheating-related variables (acceptance, expected punishments, perceived risk of detection, guilt, self-reported cheating) Cronbach’s alphas were measured.

### The individual differences in competition and the competitive climate scale

Competition-related scales were also analyzed by EFA and CFA. As Table 1 shows, IDCS and CCS had acceptable EFA results regarding total explained variance, KMO sampling adequacy and Bartlett’s test of sphericity. Furthermore, items were loaded 0.32 or higher on one factor, without higher cross-loading of 0.32. The CFA goodness of fit results were acceptable for both scales. For IDCS, CFA confirmed the three-factor structure, with SD competition, PAC, and HC emerging as distinct factors. Two factors were distinguished within the CCS: CC and DC.

**Table 1:** **Exploratory and confirmatory factor analysis results of competition scales**.

	Total explained variance (EFA; %)	KMO	Bartlett	Number of items per factor	Cronbach alphas	χ^2^/*df*	RMSEA	Robust RMSEA 90% *CI* of RMSEA	CFit	CFI	TLI
Individual differences of competition scale (IDCS)	61.35	0.865	*p* < 0.001	SD = 4	SD = 0.752	2.27	0.045	0.034–0.056	0.746	0.975	0.967
				PAC = 4	PAC = 0.823						
				HC = 4	HC = 0.769						
Competitive climate scale (CCS)	63.88	0.602	*p* < 0.001	CC = 3	CC = 0.677	1.55	0.038	0.000–0.077	0.649	0.992	0.982
				DC = 3	DC = 0.736						

*Abbreviations of the IDCS factors: SD, self-developmental competitive; PAC, positive attitudes toward competition; HC, hypercompetition. Abbreviations of the CCS factors: CC, constructive competition and DC, destructive competition. Abbreviations of the statistical measures: KMO, The Kaiser–Meyer–Olkin measure of sampling adequacy; Bartlett, Bartlett’s test of sphericity; χ^2^/*df*, chi-square degree of freedom ratio; RMSEA, root mean square error of approximation; 90% CI, RMSEA’s 90% confidence interval; Cfit, RMSEA’s test of close fit; CFI, comparative fit index; TLI, Tucker–Lewis index*.

#### The academic motivation scale

For AMS, the Guttman–Kaiser criterion indicated five factors, while scree test only indicated three. On the basis of these results, also taking into account the original theory, 3-, 4-, 5-, and 7-factor solutions were tested with EFA then CFA. The results are presented in Table 2.

**Table 2:** **Exploratory and confirmatory factor analysis results of Hungarian AMS**.

	Total explained variance (EFA; %)	KMO	Bartlett	Num. of items per factor	Cronbach alphas	Number of dropped items	χ^2^/df	RMSEA	Robust RMSEA 90% *CI* of RMSEA	CFit	CFI	TLI
Three-factor model	66.49	0.768	*p* < 0.001	TK = 3	TK = 0.793	17	2.94	0.056	0.044–0.068	0.193	0.971	0.959
				ER = 4	ER = 0.774							
				AM = 4	AM = 0.833							
Four factor model	62.98	0.837	*p* < 0.001	TK + ES = 6	TK + ES = 0.842	11	4.31	0.073	0.066–0.080	0.000	0.915	0.893
				ES = 2	ES = 0.773							
				ER + IJ = 5	ER + IJ = 0.766							
				AM = 4	AM = 0.833							
Five factor model	No solution	Ns	Ns	TK = 4	TK = 0.793	8	3.28	0.061	0.055–0.067	0.001	0.931	0.915
				ES = 4	ES = 0.737							
				IJ = 4	IJ = 0.757	
				ER = 4	ER = 0.774	
				AM = 4	AM = 0.833							
Seven factor model	No solution	Ns	Ns	TK = 4	TK = 0.793	0	3.17	0.059	0.055–0.063	0.000	0.91	0.893
				TA = 4	TA = 0.833							
				ES = 4	ES = 0.737	
				ID = 4	ID = 0.776	
				IJ = 4	IJ = 0.757	
				ER = 4	ER = 0.774	
				AM = 4	AM = 0.833	

*Abbreviations of the AMS factors: TK, intrinsic motivation to know; TA, intrinsic motivation toward accomplishment; ES, intrinsic motivation to experience stimulation; ID, identified extrinsic motivation; IJ, extrinsic motivation of introjected regulation; ER, extrinsic motivation of external regulation; AM, amotivation. Abbreviations of the statistical measures: KMO, The Kaiser–Meyer–Olkin measure of sampling adequacy; Bartlett, Bartlett’s test of sphericity; χ^2^/*df*, chi-square degree of freedom ratio; RMSEA, root mean square error of approximation; 90% CI, RMSEA’s 90% confidence interval; Cfit, RMSEA’s test of close fit; CFI, comparative fit index; TLI, Tucker–Lewis index*.

On the basis of the EFA and CFA results, the five- and seven-factor solutions did not appear to be adequate. Even after dropping items to create adequate models (containing at least three items in each factor with factor-loadings of 0.32 or above, and cross-loadings lower than 0.32; see Tabachnik and Fidell, 2001), no seven-factor solution emerged in EFA. Furthermore, for five- and seven-factors solutions, model fit indices were not acceptable by the criteria of Hu and Bentler (1999). The four-factor model provided a possible EFA solution with acceptable KMO, Bartlett test, and reliability in terms of Cronbach’s alpha. However, there were several problems with this solution. One of these factors consisted of the four items that originally belonged to TK factor and two items from the original IM/ES factor, whereas the other factor contained only two items from the original IM/ES factor (see Table 1 for abbreviations). According to Costello and Osborne (2005) two items per factor solutions is not acceptable. Furthermore, according to Hu and Bentler’s (1999) criteria, our four-factor model does not fit to the main indices (RMSEA, CI, CFit, CFI, TLI). Therefore, the four-factor solution was abandoned.

The three-factor model appeared to be adequate in terms of the EFA factor structure, KMO, Bartlett’s test of sphericity, reliability (Cronbach’s alpha), and regarding almost all of the CFA indices. On this basis, the three-factor model was chosen as the measure of the effects of EM/ER external regulation, TK, and AM on academic dishonesty. In the case of EM/ER and AM factors every original item that Vallerand et al. (1992) used were preserved. However, in order to get acceptable CFA model fit, one item (“*Because my studies allow me to continue to learn about many things that interest me*.”) was dropped from TK subscale. The deletion of this item did not change the meaning of this motivational factor, which refers to the performance of an activity for the pleasure and the satisfaction during learning.

### Descriptive statistics

Table 3 shows descriptive results for self-reported cheating and cheating-related variables (acceptance of cheating, guilt, risk of detection, and expected punishments), separately for cheating by using cheating sheets and copying from other students during the exams. More than 75% of the students used cheating sheets, and more than 60% copied during exams during the last semester. Further, for more than 50% of the respondents, both forms of cheating are acceptable, or even totally acceptable (responses 3 and 4, respectively). More than one third of the students did not feel any guilt, and another one third felt only a little guilt, after such cheating behaviors. The majority of students considered these behaviors risky, and the expected punishment for being caught cheating was either failing the exam or scolding from the teacher. Cronbach’s alphas showed good reliability regarding acceptance of cheating and guilt; however, they were only borderline in the case of risk of detection and expected punishment. Table 4 depicts means and standard deviations of Hungarian AMS, IDCS, and CC.

**Table 3:** **Percentages of students who self-reported cheating, together with cheating-related variables**.

		Self-reported cheating (%)		Acceptance	Guilt	Risk of detection	Expected punishment
Utilization of cheating sheets	No	24.6	1	4.4%	40.5%	2.4%	14.4%
			2	33.8%	37.1%	14.7%	82.6%
	Yes	75.4	3	47.1%	17.5%	72.0%	2.5%
			4	14.8%	4.9%	10.9%	0.5%
Copying from classmate during an exam	No	38.1	1	7.6%	35.3%	1.3%	12.1%
			2	39.3%	33.2%	7.8%	86.2%
	Yes	61.9	3	41.5%	21.1%	59.3%	1.2%
			4	11.5%	10.4%	31.6%	0.5%
Cronbach’s alphas				0.75	0.81	0.61	0.65

*Acceptance (1, totally unacceptable; 2, not acceptable; 3, acceptable; 4, totally acceptable); Guilt (1, not at all; 2, a little; 3, moderately; 4, very); Risk of detection (1, not at all risky; 2, not risky; 3 risky; 4 very risky); Expected punishments (1, Nothing; warning; 2, Failing on test, scolding, 3, Written warnings; 4, Severe, expelling)*.

**Table 4:** **Means and standard deviations of motivation and competition scales**.

	Academic motivation scale			Individual differences of competition scale			Competitive climate scale	
	TK	ER	AM	SD	PAC	HC	CC	DC
Likert scale	1–7	1–7	1–7	1–4	1–4	1–4	1–6	1–6
Mean	4.08	3.68	2.47	2.70	3.20	2.79	4.24	2.84
SD	1.30	1.30	1.20	0.69	0.60	0.68	0.85	1.08

*TK, intrinsic motivation to know; EMER, extrinsic motivation of external regulation; AM, amotivation; SD, self-developmental competition; PAC, positive attitudes toward competition; HC, hypercompetition; CC, constructive competitive climate; DC, destructive competitive climate*.

### Correlational results

Table 5 presents correlations between the measured variables. As expected, acceptance of cheating (*r* = 0.44, *p* < 0.001, power = 1), guilt (*r* = –0.43, *p* < 0.001, power = 1) and risk of detection (*r* = –0.23, *p* < 0.001, power = 0.96) were in stronger relationship with self-reported cheating, than with motivation [TK (*r* = –0.20, *p* < 0.001, power = 0.85); ER (*r* = 0.10, *p* < 0.05, power = 0.50; AM (*r* = 0.13, *p* < 0.001, power = 0.51)] and competition-related variables [SD (*r* = 0.02, *p* = 0.713, power = 0.73); PAC (*r* = 0.01, *p* = 0.762, power = 0.77); AC (*r* = 14, *p* < 0.001, power = 0.55); DC (*r* = 0.00, *p* = 0.95, power = 0.95); CC (*r* = –0.09, *p* = 0.093, power = 0.50)]. Furthermore, on the basis of the power analysis, extrinsic motivation was not reliably linked to self-reported cheating, nor correlated with the cheating-related variables [acceptance (*r* = 0.02, *p* = 0.695, power = 0.71), guilt (*r* = 0.00, *p* = 1, power = 1), risk of detection (*r* = 0.04, *p* = 0.33, power = 0.52), or with punishment (*r* = 0.00, *p* = 0.96, power = 0.96)].

**Table 5:** **Descriptive statistics: correlations between the measured variables**.

Variable	1	2	3	4	5	6	7	8	9	10	11	12
**CHEATING VARIABLES (*N* = 620)**												
1. Self-reported cheating	–											
2. Acceptance of cheating	0.44*** (1.00)	–										
3. Guilt	−0.43*** (1.00)	−0.54*** (1.00)	–									
4. Risk of detection	−0.23*** (0.96)	−0.29*** (1.00)	0.30*** (1.00)	–								
5. Expected punishment	−0.10* (0.50)	−0.13*** (0.46)	0.12** (0.49)	0.25*** (0.99)	–							
**ACADEMIC MOTIVATION (*N* = 620)**												
6. IM to know	−0.20*** (0.85)	−0.29*** (1.00)	0.33*** (1.00)	0.14*** (0.37)	0.06 (0.51)	–						
7. EM external regulation	0.10* (0.50)	0.02 (0.71)	0.00 (1.00)	0.04 (0.52)	0.00 (0.96)	0.08 (0.51)	–					
8. Amotivation	0.13*** (0.51)	0.25*** (0.99)	−0.25*** (0.99)	−0.17*** (0.66)	0.01 (0.89)	−0.30*** (1.00)	−0.15*** (0.44)	–				
**COMPETITION IND. DIFF. (*N* = 620)**												
9. Self-developmental competition	0.02 (0.73)	−0.01 (0.88)	0.07 (0.50)	0.06 (0.50)	0.08* (0.50)	0.30*** (1.00)	0.14*** (0.36)	−0.07 (0.51)	–			
10. Positive attitudes towards competition	0.01 (0.77)	−0.08 (0.50)	0.12** (0.48)	0.04 (0.53)	0.04 (0.53)	0.21*** (0.90)	0.16*** (0.76)	−0.12** (0.48)	0.58*** (1.00)	–		
11. Hypercompetition	0.14*** (0.55)	0.14*** (0.53)	−0.16*** (0.54)	−0.06 (0.50)	−0.07 (0.50)	−0.09* (0.50)	0.12** (0.50)	0.18*** (0.71)	0.19*** (0.82)	0.26*** (1.00)	–	
**COMPETITIVE CLIMATE (*N* = 381)**												
12. Destructive competition	0.00 (0.95)	−0.03 (0.62)	0.01 (0.91)	–0.12* (0.50)	0.05 (0.53)	0.02 (0.75)	0.06 (0.51)	0.00 (0.93)	−0.05 (0.52)	0.00 (0.96)	0.04 (0.56)	–
13. Constructive competition	−0.09 (0.50)	−0.10* (0.50)	0.08 (0.50)	0.04 (0.54)	0.01 (0.82)	0.07 (0.50)	0.01 (0.89)	−0.09 (0.50)	−0.07 (0.50)	0.00 (0.93)	−0.07 (0.50)	0.02 (0.67)

*p < 0.05. **p < 0.01. ***p < 0.001. Achieved powers are in bracelets.

Similar patterns were revealed for correlations between self-reported cheating and the IDCS variables of SD and PAC and both of the CCS variables (CC, DC). Hypothesis 1 was partly confirmed, because these competition-related factors were not strongly linked to self-reported cheating, and they had only weak correlations with the cheating-related variables. On the other hand, the TK, AM, and IDCS/HC factors were correlated with self-reported cheating as the required power exceeds to lower threshold of 0.8. However, out of the eight motivational and competition-related variables, TK was the only one, which was in reliable relationship with self-reported cheating behavior, which partly confirms the second hypothesis concerning the distinct effect of competition- and motivation-related variables. TK was also reliably related to acceptance of cheating (*r* = –0.29, *p* < 0.001, power = 1) and guilt feeling after cheating (*r* = 0.33, *p* < 0.001, power = 1). A similarly reliable, but opposite direction relationship, was obtained for AM with acceptance (*r* = 0.25, *p* < 0.001, power = 0.99) and guilt feeling (*r* = –0.25, *p* < 0.001, power = 0.99). In sum, among motivation and competition-related variables only intrinsic motivation can be significantly and reliably linked to self-reported cheating, with AM, also showing reliable relationship with the acceptance and guilt factors. The correlation of self-reported cheating and its related variables with the other motivational and competition-related factors (ER, SD, PAC, HC, DC, CC) was not reliable, as was shown by the power analysis. These results partly confirm our first and second hypotheses.

After measuring the correlations between the examined variables we were interested in the comparison of the strength (by using Fisher *r*-to-*z* transformation) of these relationships in order to test the third hypothesis. According to this hypothesis the strength of motivational and competition-related variables (individual differences and climate) was less related to self-reported cheating than other relevant variables as acceptance of cheating, guilt after cheating, risk of detection, or expected punishments. Table 6 contains the results of the comparisons between correlational coefficients regarding self-reported cheating. The results suggest that acceptance of cheating and guilt were in the strongest relationship with cheating: in comparison with all of the motivational (TK, ER, AM) and competition-related variables (SD, HC, PAC, CC, DC) guilt and acceptance of cheating have stronger relationship with self-reported cheating. The link between risk of detection and self-reported cheating in four out of eight cases was stronger, than the relationship between self-reported cheating and motivational and competition-related variables. Whereas there was no significant difference in the other four cases regarding the correlational coefficients. However, motivational and competition-related variables were similarly related to self-reported cheating as expected punishments. In sum, results confirmed the third hypothesis, because acceptance of cheating, guilt, and risk of detection were in stronger relationship with self-reported cheating than motivational and competition-related variables.

**Table 6:** **Differences between correlational coefficients of self-reported cheating and the examined variables**.

Variable	Fisher *r*-to-*z* transformations in the case of self-reported cheating										
	1	2	3	4	5	6	7	8	9	10	11
**CHEATING-RELATED VARIABLES (***N*** = 620)**											
1. Acceptance of cheating	–										
2. Guilt	0.22	–									
3. Risk of detection	4.18***	3.96***	–								
4. Expected punishment	6.53***	6.32***	2.35*	–							
**ACADEMIC MOTIVATION (***N*** = 620)**											
5. IM to know (TK)	4.73***	4.52***	0.55	1.8	–						
6. EM external regulation (ER)	6.53***	6.32***	2.35*	0.00	1.8	–					
7. Amotivation (AM)	6.00***	5.78***	1.82	0.53	1.26	0.53	–				
**COMPETITION INDIVIDUAL DIFF. (***N*** = 620)**											
8. Self-developmental competition (SD)	7.94***	7.73***	3.76***	1.41	3.21**	1.41	1.95	–			
9. Positive attitudes toward competition (PAC)	8.12***	7.90***	3.94***	1.59	3.39***	1.59	2.12*	0.18	–		
10. Hypercompetition (HC)	5.82***	5.6***	1.64	0.71	1.09	0.71	0.18	2.12*	2.30*	–	
**COMPETITIVE CLIMATE (***N*** = 381)**											
11. Destructive competition (DC)	7.23***	7.04***	3.59***	1.54	3.10**	1.54	2.00*	0.31	0.15	2.16*	–
12. Constructive competition (CC)	5.85***	5.66***	2.2	0.15	1.72	0.15	0.62	1.08	1.23	0.78	1.24

*p < 0.05. **p < 0.01. ***p < 0.001.

### The relationship pattern of motivation, competition, and academic cheating

No previous studies examined the impact of motivations and competition-related variables on self-reported cheating in the context of other cheating-related variables in a path model. Therefore, the present path-analysis is basically exploratory. We intended to explore how competition and motivation-related variables are connected with cheating-related variables and in which ways they influence directly or indirectly cheating. Consequently, no pre-existing theoretical background was available. However, on the basis of the correlations, we expected that the path model would shed light on (1) different patterns of influence of motivational vs. competitive factors, (2) their smaller of influence on cheating in comparison with other already examined variables as acceptance of cheating, risk of detection, and expected punishments.

Structural equation modeling (SEM) was used to explore the relationship pattern of self-reported academic cheating, motivational (TK, ER, AM), individual, and situational competition-related variables (SD, PAC, HC, CC, DC), and cheating-related variables (acceptance, guilt feeling, risk of detection, and expected punishment) and the observable variable GPA. Self-reported academic cheating behavior was determined on the basis of two variables: use of cheating sheets during a test and copying from a classmate during a test. The latent variables of acceptance, guilt feeling, risk of detection, and expected punishments were addressed by the variables from these two types of cheating situations. GPA was used as an observed variable, while the latent variables regarding competition (SD and HC) and academic motivation (intrinsic, extrinsic, and AM) was determined with the items showing the best fit in the CFA models.

**Figure 1:**
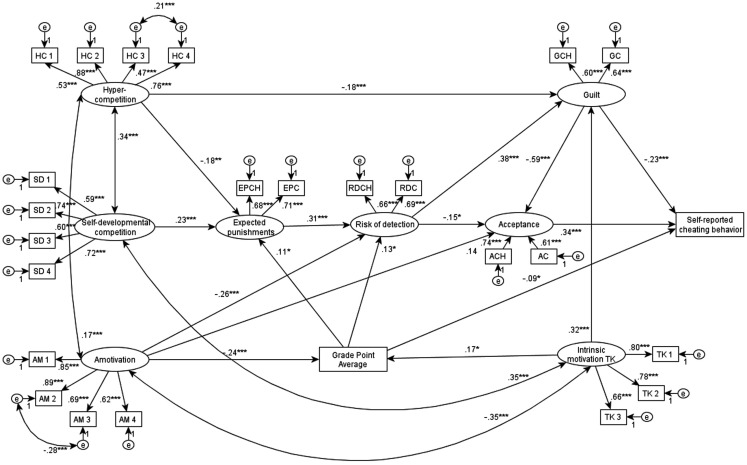
**Exploratory path-analysis of the examined variables**. Associations (standardized path coefficients β) among TK, amotivation, HC, SD, risk of detection, GPA, expected punishments, acceptance of cheating, guilt, and self-reported academic cheating. HC, items from hypercompetition scale; SD, items from self-developmental competition scale; AM, items from amotivation factor; TK, items from intrinsic motivation to know factor; EPCH, expected punishments for using cheating sheets, EPC, expected punishments for copying; RDCH, risk of detection for using cheating sheets; RDC, risk of detection for copying; ACH, acceptance of cheating sheets; AC, acceptance of copying; GCH, guilt after using cheating sheets; GC, guilt feeling after copying; e, error; χ^2^(254, *N* = 620) = 453.769, *p* < 0.001 (χ^2^/*df* = 1.786), CFI = 0.959, TLI = 0.952, RMSEA = 0.036, $R_{\text{self-reported cheating behaviour}}^{2}=0.29$, $R_{\text{Acceptance of cheating}}^{2}=0.53$, $R_{\text{Guilt}}^{2}=0.32$, $R_{\text{GPA}}^{2}=0.11$, $R_{\text{Risk of detection}}^{2}=0.22$, $R_{\text{Expected punishment}}^{2}=0.07.$ *p ≤ 0.05. **p ≤ 0.01. ***p ≤ 0.001. Extrinsic motivation, positive attitudes toward competition, constructive and destructive competitive climate scales are not part of the model.

Several models were tested. Here only the final and best fitting model is presented, but alternative models are available upon request. Figure 1 depicts the fitted model with standardized estimates. According to the final model [χ^2^(254, *N* = 620) = 453.769, *p* < 0.001 (χ^2^/*df* = 1.786), CFI = 0.959, TLI = 0.952, RMSEA = 0.036] the direct effect of three variables to self-reported cheating behavior (*R*^2^ = 29.0%) appeared: (a) acceptance (β = 0.34, *p* < 0.001), (b) guilt (β = −0.23, *p* < 0.001) and (c) GPA (β = −0.09, *p* < 0.05). Furthermore, the cheating-related variables were interconnected. Motivational and competition-related factors were indirectly linked with self-reported cheating through the variables related to cheating. HC had a positive effect on cheating, mediated by punishment (β = −0.18, *p* < 0.01) and guilt (β = −0.18, *p* < 0.001), whereas SD competition was in positive relationship (β = 0.26, *p* < 0.001) with expected punishments, thus having an indirect negative effect on cheating. These results confirm our first hypothesis. TK was positively related to guilt (β = 0.32, *p* < 0.001) and it also influenced positively GPA (β = 0.17, *p* < 0.001). According to the model, AM was negatively related to the perceived risk of detection (β = −0.26, *p* < 0.001) and GPA (β = −0.24, *p* < 0.001), while it had positive effect on acceptance of cheating (β = 0.14, *p* < 0.001). These results confirm our second hypothesis. In sum, competition-related variables (SDC, HC) were in relatively weak and mediated relationship with self-reported cheating. Furthermore, the model’s results suggest that AM was one of the main motivational variables responsible for cheating. However, intrinsic motivation (TK) can prevent cheating through expected guilt and GPA. In this model, four covariances between variables and two error covariances appeared.

## Discussion

The present study aimed to measure the effect of academic motivation, individual differences in competition and competitive climate on academic cheating. Results partly confirmed our first hypothesis, in which competition was assumed to have a multifaceted impact on cheating behavior. According to the correlational and SEM results, PAC, and both constructive and destructive competitive climate are unrelated to academic dishonesties. SD competition is not reliably related to self-reported cheating and in the SEM model it has negative indirect effect on cheating through several factors. HC is in a weak, significant but not reliable relationship with self-reported cheating, acceptance of cheating and guilt, and in the SEM model it is indirectly positively linked to cheating. In sum, our hypotheses regarding individual differences of competition were partly confirmed; however, hypotheses of competitive climate were not.

Our second goal was to distinguish the effects of motivation vs. competition-related factors of cheating. Regarding motivational issues intrinsic motivation and AM are reliably linked to cheating, and in the SEM model both influence it indirectly. Intrinsic motivation negatively related to self-reported cheating, acceptance and positively to guilt. AM has an inverse pattern: it is reliably and positively linked to acceptance of cheating and negatively related to guilt. Furthermore, in the SEM model AM has a positive effect on self-reported cheating through perceived risk of detection, acceptance of cheating and GPA. Extrinsic motivation was not reliably related to cheating. Whereas, according to the correlational and SEM results, several motivational and competition-related variables are not in a significant relationship with cheating, such as PAC, constructive and destructive competitive climate. Moreover, as Figure 1 shows, in the SEM model, HC, and SD competition have a different mediated effect pattern on self-reported cheating than extrinsic motivation or AM.

In the case of academic motivation, it is important to mention that, on the basis of the results, it is not the extrinsic motivation, which can be accounted for cheating, but AM. These results are in accordance with Angell’s (2006) findings, which showed that AM is in a positive correlation with the frequency of cheating. Furthermore, the effect of intrinsic motivation on self-reported cheating is relatively high. AM seems to be a more important aspect of motivation, which can induce cheating in a larger extent, compared to extrinsic motivation. Furthermore, on the basis of the model, intrinsic motivation has a larger effect on cheating than extrinsic motivation. It has several theoretical and practical implications. Altogether, these results suggest that it can be more important to build intrinsic motivation and eliminate AM, rather than to reduce extrinsic motivation in order to decrease the prevalence of academic dishonesties. In terms of practical perspectives, in the case of the students who are extrinsically motivated, the best way to prevent them from cheating is not necessarily the reduction of their extrinsic motivation; it can be better to increase their intrinsic motivations.

Results of competition-related variables may provide new perspectives to reinterpret former relevant studies (Taylor et al., 2002; Anderman and Murdock, 2007). First, it is possible to distinguish hyper, SD competition, and PAC; second, constructive and destructive competitive climates should be taken into account. Consequently, similarly to Ryckman et al.’s (1990, 1996), Tjosvold et al.’s (2003, 2006), and Fülöp’s (2008) distinction, it is important to examine different forms of competition, both at the individual level and at the contextual level. Another important finding reflects the relationship between motivation and competition. Previously (Anderman and Murdock, 2007), extrinsic motivation and performance goal orientation were interpreted as phenomena which go hand in hand with competition and which are reliable predictors of cheating. Nevertheless, our results suggest that there are correlations between academic motivations and individual differences of competition (see Table 4); these competition-related and motivational factors have dissimilar effects on academic cheating.

Finally, our third hypothesis was confirmed: motivational and competition-related variables that showed significant relationship have smaller effects than such factors as acceptance or guilt. In the SEM path model, motivational and competition-related personality characteristics have a mediated effect on cheating. They influence in a weaker manner cheating compared to other variables, such as acceptance of cheating, guilt or risk of detection. Consequently, these results suggest that individual factors of motivation and competition have a slight effect on cheating in comparison with other more proximal variables. Moreover, competitive climate is unrelated from self-reported exam-cheating.

These results might be explained in different ways. Several reasons can be taken into consideration regarding the minor role of motivational and competition-related individual factors. The first explanation can derive from the high frequency of cheating: more than 60% of the students who responded used cheating sheets or copied at least once during the last semester. In schools, in which cheating rates are lower, motivational and competition-related individual factors may have a larger impact on cheating. The second explanation refers to McCabe and Trevino’s (1993, 1997) results from large scale studies, along with Whitley’s (1998) meta-analysis, and with Anderman and Murdock’s (2007) textbook, situational and interpersonal factors can be accounted to a larger extent for the presence of cheating in comparison with individual factors, as motivation and personality differences in competition. However, regarding the effects of competitive climate, this situation-focused explanation might not be true. This explanation could be supported if, similarly to Whitley’s (1998) meta-analysis, we found that competitive climate is in a moderate relationship with cheating. However, according to our study’s results, it was not the case: neither constructive, nor destructive competitive climate are linked to cheating.

It is important to mention several limitations in this study. Cheating was measured in a dichotomous way (cheated or not) in two situations. Therefore, this dependent variable was based on a three-point scale: (no cheating, cheated in only one way – copying or using cheating sheets, cheated in both ways). Asking cheating occurrences in a more sensitive scale and/or use more situations could have lead to more precise measurement. Furthermore, it would be useful examine the utilized competition-related scales in other studies. In spite of the appropriate validity and reliability in terms of EFA, CFA and Cronbach’s alphas, further construct, convergent, discriminant, predictive validity, and test-retest examinations would be required for future studies.

Moreover, several popular forms of academic cheating were not analyzed, for example plagiarism or the use of electronic devices in order to cheat. Beyond these problems, in the case of cheating-related variables (self-reported cheating, acceptance of cheating, guilt feeling, risk of detection, and expected punishments) distribution was not normal. We did not make transformations in order to obtain the necessary normal distribution concerning these variables because our goal was to preserve the characteristics of Hungarian students in this field.

Furthermore, even if previous studies could suggest the seven factor structure (Fairchild et al., 2005; Barkoukis et al., 2008; Smith et al., 2010), in our study, we could only identify three of Vallerand et al.’s (1989, 1992, 1993) AMS’ factors. Concerning the impact of motivational factors on academic dishonesties, the seven factor solution could lead to more refined results, because in this way the effect of more or less controlled and autonomous forms of extrinsic motivations could have been examined.

Further investigations should not only use the theoretical and methodological framework of intrinsic and extrinsic motivation, but achievement goal theories. Future studies carried out with Patterns of Adaptive Learning Survey (PALS – Midgley et al., 2000) or the revised version of Achievement Goal Questionnaire (AGQ-Revised – Elliot and Murayama, 2008) could contribute to the deeper understanding of the relationship between students’ achievement goals and cheating behavior.

The next limitation concerns the reliability of self-reported data. On the basis of our previous results, self-reported data can be dissimilar from experimental behavioral data (Orosz, 2010). Therefore, it would be important to see how competition-related individual differences influence cheating behavior, and how constructive vs. DC affect dishonest exam conduct.

We have to mention here that, dissimilarly to previous USA studies, our results are in accordance with numerous other studies from post-socialist countries, which find high cheating rates in this region (Poltorak, 1995; Lupton et al., 2000; Magnus et al., 2002; Grimes, 2004; Hrabak et al., 2004; Teixeira and Rocha, 2006, 2010; Orosz, 2009). It is possible that, in such countries, in which cheating tends to occur frequently, motivational and competition-related issues in cheating might be less important. Even if students state that they felt guilty, if they did not accept cheating and if they perceived these behaviors as dangerous, they may have more possibilities to cheat in comparison with other countries or other educational systems, in which cheating is more intensively regulated by honor codes (McCabe and Trevino, 1997; McCabe et al., 1999). Therefore, it would be interesting to estimate these factors’ effect on cheating in such educational systems in which cheating rates are lower and in which students have less possibility to cheat.

Finally, these results suggest that Anderman and Murdock (2007) seem to be less accurate in the Hungarian educational context, by claiming that competition is a reliable predictor of cheating. Taking into account the present sample, neither constructive, nor DC can be accounted for high level of cheating. Furthermore, SD competitive traits are indirectly and negatively linked to cheating, and only HC is in a positive (but not reliable) relationship with cheating through several cheating-related variables. Consequently, it is important to take into account the fact that competition does not have inherently a positive impact on cheating, as, its distinct individual and situational forms can be positively, negatively linked to academic cheating or unrelated to it. Several studies (i.e., Fülöp, 2008) showed multiple facets of competition; researches have found positive forms of competition since the beginning of the twentieth century (Hurlock, 1927; Erev et al., 1993; Tassi and Schneider, 1997; Hawley, 2003, 2006; Tjosvold et al., 2006). Therefore, accusing competition as a holistic phenomenon that reliably predicts cheating can be misleading.

Previous studies from the USA, Singapore, and Ethiopia (Smith et al., 1972; Lim and See, 2001; Teferra, 2001; Taylor et al., 2002; Anderman and Murdock, 2007) blame competition to be a predictor of cheating, while other studies from post-socialist countries claim that it is the lack of competition which induces cheating (Poltorak, 1995; Magnus et al., 2002). In order to explore these inconsistencies, it would be fruitful to examine the effect of competition both at the individual level and at the situational-level in different cultures. Furthermore, in these researches it would be necessary to put emphasis on the different forms of cheating. Maybe individual forms of cheating occur more frequently in highly competitive educational systems, in which students’ individual achievement is evaluated frequently. Whereas collaborative forms of cheating can be more frequent in such educational contexts in which competition is less intense and/or students are evaluated on the basis of their collaborative achievements.

## Conclusion

The present study aimed to measure the relationship patterns of academic motivations, individual differences in competition, and competitive climate on academic cheating among Hungarian high school students. The results suggest that neither constructive, nor destructive competitive climate is related to students’ self-reported cheating behavior. Furthermore, while PAC do not have an effect on cheating, SD competition has a negative effect, and HC personality traits are in a weak and unreliable positive relationship with self-reported test cheating. Moreover, intrinsic academic motivation is negatively linked to acceptance of cheating and positively linked to guilt after cheating, and it has a negative indirect effect on self-reported cheating. Whereas an inverse relationship pattern was revealed in the case of AM: positive relationship with acceptance of cheating, negative with guilt and indirect positive effect on self-confessed exam-cheating. In comparison with intrinsic motivation and AM, the role of extrinsic motivation in academic cheating is less significant. However, other more proximal variables, such as acceptance of cheating, guilt, and GPA have a direct and significant impact on self-reported cheating.

Six main conclusions can be drawn from the results: (a) It is important to distinguish different forms of competition, both at the individual level and at the contextual level in order to explore its impact on cheating. (b) The individual level of competition-related and academic motivational factors cannot be dealt with as interconnected phenomena because they have different patterns of effect on cheating. (c) SD competitive attitudes negatively influence cheating, whereas hypercompetitive traits have a positive impact. (d) The role of extrinsic motivation is less important than the role of intrinsic motivation and AM. (e) Variables, such as acceptance of cheating and guilt, have larger and more direct impact than competition-related variables or extrinsic motivation. Very probably, at least in Hungary, not all forms of competition are toxic ingredients in a classroom, and not all of them are reliable predictors of cheating.

## Conflict of Interest Statement

The authors declare that the research was conducted in the absence of any commercial or financial relationships that could be construed as a potential conflict of interest.

## Supplementary Material

The Supplementary Material for this article can be found online at http://www.frontiersin.org/Educational_Psychology/10.3389/fpsyg.2013.00087/abstract

## Footnotes

^1^However, it is important to mention that extrinsic motivation can be induced by many other factors beyond competition, because external goals can be set not only in negative interdependent situations in which someone has to win and someone has to lose.
